# Uncoupling Protein 2 Drives Myocardial Dysfunction in Murine Models of Septic Shock

**DOI:** 10.1155/2019/9786101

**Published:** 2019-04-04

**Authors:** Rong Tang, Ping-ping Qi, Yan-song Liu, Liu Jia, Rui-jin Liu, Si-cong Wang, Chang-song Wang, Yang Gao, Hong-liang Wang, Kai-jiang Yu

**Affiliations:** ^1^Department of Critical Care Medicine, The Second Affiliated Hospital of Harbin Medical University, 246 Xuefu Road, Harbin, 150086, Heilongjiang Province, China; ^2^Departments of Blood Transfusion, The First Affiliated Hospital of Harbin Medical University, Heilongjiang Province, Harbin, China; ^3^Department of Critical Care Medicine, The Cancer Hospital of Harbin Medical University, 150 Haping Road, Harbin, 150081, China; ^4^Institute of Critical Care Medicine, Heilongjiang Academy of Medical Science, 150 Haping Road, Harbin, 150081, China; ^5^Department of Critical Care Medicine, The First Affiliated Hospital of Harbin Medical University, Harbin 150001, China

## Abstract

Cardiac dysfunction is a major component of sepsis-induced multiorgan failure in critical care units. Uncoupling protein 2 (UCP2) involves immune response, regulation of oxidative stress, and maintenance of mitochondrial membrane potential as well as energy production. However, whether and how UCP2 plays roles in the development of septic cardiac dysfunction are largely unknown. Here, intraperitoneal injection of LPS significantly activated UCP2 expression accompanied by a significant decrease of cardiac function and caused a significantly lower survival rate in mice. Of note, knockdown of UCP2 through a cardiotropic adenoassociated viral vector carrying a short hairpin RNA (shRNA) specifically targeting the UCP2 evoked resistance to LPS-triggered septic cardiac dysfunction and lethality* in vivo*. Moreover, UCP2 deficiency ameliorated the reduced levels of intracellular ATP in the LPS-challenged heart tissues and preserved mitochondrial membrane potential loss in primary adult mouse cardiomyocytes in LPS-challenged animals. Mechanistically, we confirmed that the inhibition of UCP2 promoted autophagy in response to LPS, as shown by an increase in LC3II and a decrease in p62. At last, the autophagy inhibitor 3-MA abolished UCP2 knockdown-afforded cardioprotective effects. Those results indicate that UCP2 drives septic cardiac dysfunction and that the targeted induction of UCP2-mediated autophagy may have important therapeutic potential.

## 1. Introduction

Sepsis is a systemic inflammatory response syndrome caused by infection, which is a leading cause of death worldwide [1–3]. Cardiac dysfunction is a well-known serious component of the multiorgan failure associated with sepsis. However, the underlying mechanisms of myocardial depression of sepsis are not yet known.

Mitochondria comprised about 30% of myocardial volume and work cooperatively to provide energy, which in cardiomyocytes is an important target in septic myocardial injury. With the development of concepts of bioenergetics and disease-caused cell hypoxia, it is hypothesized that mitochondrial energy metabolism is associated with its own dynamic changes [4, 5]. Mitochondrial ATP and ROS production are regulated by a family of uncoupling proteins (UCPs), which are located in the mitochondrial inner membrane and catalyze a regulated proton leak from the intermembrane space into the mitochondrial matrix [6, 7]. Thus far, several UCP family members have been discovered [8]. UCP1 is predominantly expressed in brown adipose tissue which plays important role in thermogenesis [9]. UCP2 is widely expressed in various tissues, while UCP3 is predominantly expressed in skeletal muscle and BAT and present in the heart albeit at lower levels compared with skeletal muscle [10]. UCPs are present on the mitochondrial inner membrane16. An elevated level of UCPs can cause heart failure or myocardial injury. Numerous studies showed that UCP2 involved the regulation of inflammation, regulation of oxidative stress, maintenance of mitochondrial membrane potential, and energy production, which may be related to the pathophysiology of sepsis [11, 12]. However, the role of UCP2 in sepsis and the underlying mechanisms remain to be further explored.

Autophagy contributes to the regulation and function of diverse range of cellular responses, such as metabolic balance [13], cell fate [14], and inflammation [15]. Under normal physiological responses or mild stress, autophagy provides a level of control to promote survival and is therefore adaptive. However, under severe or chronic stress, either excessive autophagic activity or inadequate autophagy can lead to massive self-degradation or the accumulation of toxic materials, respectively; either of these outcomes is maladaptive and can ultimately cause cell death [16]. Clinical and preclinical studies indicate that sepsis triggers autophagy in multiple organs including the heart [17, 18]. However, our current understanding of the role of autophagy in the pathogenesis of sepsis remains limited and inconclusive. Given that UCP2 is a regulator of mitochondrial potential [19, 20], we tested whether the redundancy of myocardial UCP2 alters autophagy, leading to the adaptive or maladaptive effects.

Our studies were performed using a mouse model of sepsis induced by LPS, a known toxic component of gram-negative bacteria. The investigation, summarized in this study, was aimed at addressing level of UCP2 in myocardium of LPS-challenged mice and the role and underlying molecular basic of UCP2 during septic cardiac dysfunction.

## 2. Materials and Methods

### 2.1. LPS-Induced Sepsis Model

10-week-old mice were injected intraperitoneally with LPS (25 mg/kg,* Escherichia coli* 0111: B4, Sigma-Aldrich) dissolved in PBS or PBS alone as their control mice as described previously. Survival rate was then recorded every 3 h for a 60-h period. At 12 h after injection, the blood sera and heart tissues were collected and stored at −80°C for protein and RNA analysis. The procedures were approved by the Institutional Animal Care and Use Committee and Ethics Committee of the Harbin Medical University and complied with the standards of animal welfare in China.

### 2.2. Cardiac Function Assessment by Echocardiography

Mice were anesthetized after 12 h LPS challenge by isoflurane inhalation and echocardiographic assessments were examined using the Vevo 2100 system (Visual Sonics, Toronto, Canada) as previously described. Heart rate was maintained at 450–550 bpm. The mitral valve leaflet was visualized and its function was assessed at long axis B-mode view by placing the transducer on the left lateral chest wall. End-systolic and end-diastolic LV dimensions and wall thicknesses were measured according to the American Society of echocardiography guidelines as applied to mice. LV cavity size and wall thickness were measured in at least 3 beats from each projection and averaged. LV functional indexes were calculated from the M-mode measurements.

### 2.3. Cardiomyocyte Culture and Treatment

Mouse hearts were removed after 12 h LPS challenge and perfused using a Langendorff system for ~3 minutes with a Ca^2+^-free bicarbonate-based buffer. Enzymatic digestion was initiated by adding collagenase type B/D to the perfusion solution. After approximately 7 minutes, the left ventricle was removed and discarded. Cardiomyocytes (CMs) from male mice (8–10 weeks) in the left ventricular wall were isolated as we previously reported, and plated at 1–2 × 10^4^ cells/cm^2^ in mouse laminin precoated culture dishes. This technique results in > 95% pure isolated CMs. The cellular survival rate was > 80% at 12 h and exceeded 65% after 24 h of isolation. ΔΨ_m_ was detected as described previously. The cultured cardiomyocytes were incubated with tetramethylrhodamine ethyl ester (TMRE, 50 nmol/L, Molecular Probes) for 20 min. After loading, the cells were washed twice with Krebs–Henseleit solution. The cardiomyocytes attached to the coverslips were then examined with Leica TCS SP2 confocal laser scanning microscope.

### 2.4. ATP Measurements

ATP content of heart tissues was measured with an ATP Bioluminescent Assay kit (Beyotime Biotechnology) according to the manufacturer's instructions.

### 2.5. Western Blot

Mouse left ventricles were lysed with RIPA buffer with protease inhibitors followed by centrifuging at 13,000 g for 10 min [21]. The concentration of proteins was determined using the Bradford method. The antibody information used in this study is as follows: UCP2 (1:2000) beclin 1 (1:2000), anti-GAPDH (1:8000,), anti-LC3 (1:2000), anti-SQSTM1 (P62, 1:5000), anti-AMPK (1:2000), anti-p-AMPK T172 (1:2000), anti-ULK1 (1:2000), and anti-p-ULK1 S317 (1:1000). Antibodies were purchased from Cell Signaling or Sigma. The experiments are repeated at least 3 times and the images were taken with an enhanced chemiluminescence (ECL) reagent.

### 2.6. Quantification of mRNA by Reverse Transcriptase Quantitative PCR

SYBR green (TOYOBO) real-time PCR (Q-PCR) for UCP2 was performed using cDNA generate fdrom total RNA extracted from LV tissue. UCP2 primers are as follows: sense, 5′-ATGGTTGGTTTCAAGGCCACA-3′ and antisense, 5′- TTGGCGGTATCCAGAGGGAA-3′.

### 2.7. Construction of Recombinant AAV9 with UCP2-shRNA

Three shRNA sequences specifically targeting on UCP2 were designed. The inhibition efficiency of the three UCP2-shRNA sequences was tested in cell line. The UCP2-shRNA sequence that achieved the greatest inhibition of UCP2 expression was chosen for the in vivo study. This sequence was designed to target on mouse UCP2 at 5′- GGAAAGGGACTTCTCCCAATG-3′. This UCP2-shRNA was then constructed into AAV9 vector under the control of RNA polymerase III promoter U6. AAV9.U6-UCP2-shRNA plasmid DNA was then packaged with pHelper and pAAV-RC to produce recombinant AAV9-shUCP2. The titer was determined by real-time PCR. AAV9 with a scrambled shRNA sequence was also constructed and served as a control (AAV9-NC). Physiological serum solution (100 *μ*L) containing 3 × 10^11^ genome copies per 1 mL AAV9 was injected intravenously into tail veins of 4-week-old male mice. An AAV9 encoding shRNA specific for UCP2 (AAV9-shUCP2) or its control was injected intravenously into tail veins of mice, and 4 weeks later, LPS injection was performed.

### 2.8. Statistical Analysis

Dates were presented as the means ± SEM of at least three independent experiments. Survival data were used to construct Kaplan-Meier curves and analyzed by the Logrank test. After confirming that all variables were normally distributed by the Kolmogorov-Smirnov test followed by Q-Q plots analysis, statistical differences were determined by Student's t-test for comparison between two groups and ANOVA followed by Bonferroni's multiple comparison test for comparison among three or more groups.* P* values of less than 0.05 were considered statistically significant. Statistical calculations were carried out using GraphPad Prism 6.0.

## 3. Results

### 3.1. UCP2 Was Upregulated during LPS-Induced Sepsis

**Figure 1:**
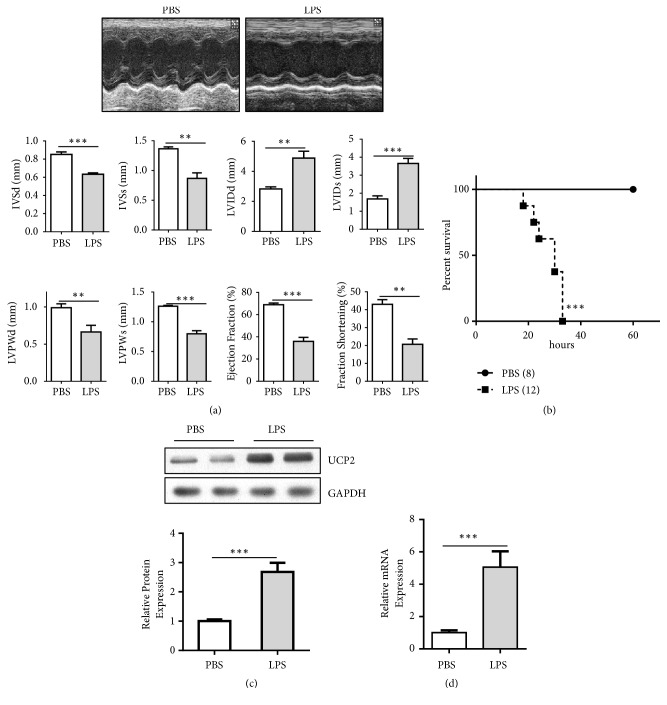
UCP2 is activated in murine septic hearts. (a) Representative images (upper panel) and results of echocardiographic (lower panel) assessment 12 h after intraperitoneal injection of PBS or LPS in mice (n = 5 for each group). (b) Survival of WT mice intraperitoneally injected with PBS or LPS, monitored every 3 h for a 60-h period (n = 8 for PBS, n = 12 for LPS). (c) Western blot detection of UCP2 protein levels in cardiac tissues 12 h after intraperitoneal injection of PBS or LPS in WT mice (n = 5 for each group). (d) Real-time PCR analysis of UCP2 protein levels in cardiac tissues 12 h after intraperitoneal injection of PBS or LPS in WT mice (n = 3 for each group). Data were presented as means ± SEM. ^*∗∗∗*^*P*<0.001 vs. the indicated group. IVSd, interventricular septum thickness at end-diastole; IVSs, interventricular septal thickness at end-systole; LVIDd, left ventricular internal diastolic diameter; LVIDs, left ventricular internal systolic diameter; LVPWd, left ventricular diastolic posterior wall thickness; LVPWs, left ventricular systolic posterior wall thickness.

We examined the expression levels of UCP2 to explore its potential role in the process of septic cardiac dysfunction. LPS or an equal volume of PBS was intraperitoneally injected into the mice. Cardiac function was assessed by echocardiography 12 hours after injection, and survival was monitored every 3 hours for 60 hours. Predictably, there is remarkably decrease of cardiac function followed by lower survival rate with LPS injection (Figures 1(a) and 1(b)).

Next, we examined the UCP2 expression in myocardium. Western blot analysis as well as real time PCR showed upregulation of UCP2 protein in the LPS-insulted septic hearts (Figures 1(c) and 1(d)). Taken together, our data indicate that UCP2 is an effector of septic cardiac dysfunction.

### 3.2. Knockdown of UCP2 Improved Cardiac Outcomes in LPS-Induced Sepsis

**Figure 2:**
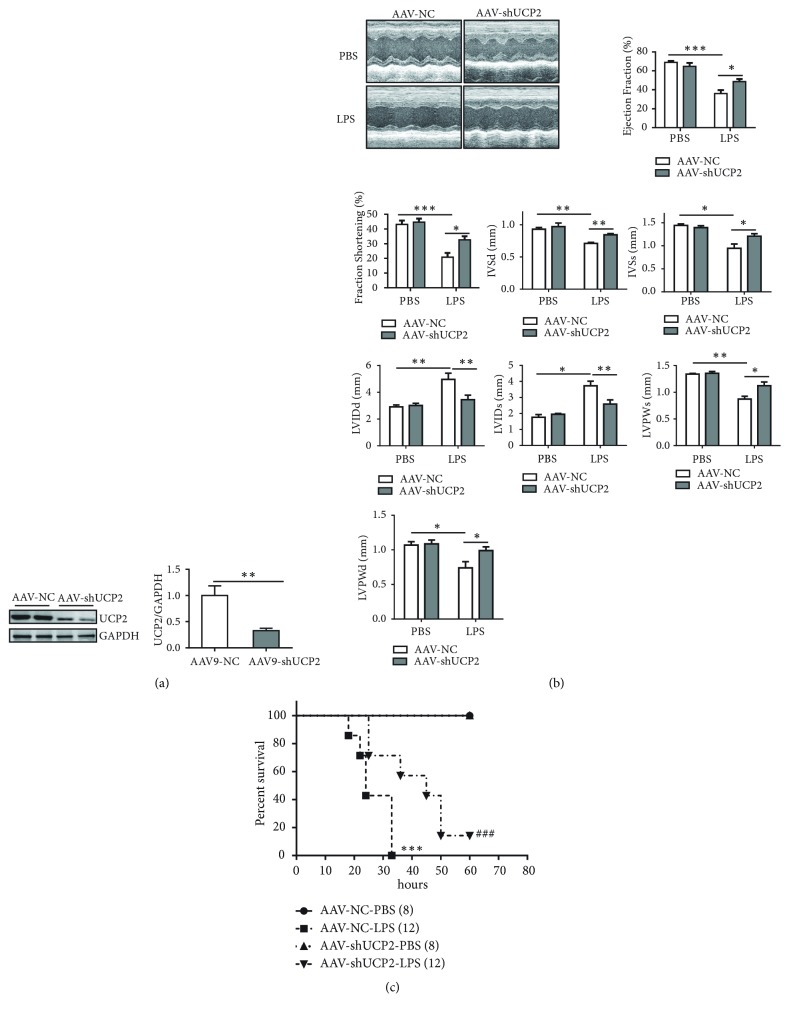
UCP2 knockdown resists septic cardiac dysfunction. (a) Western blot analysis of knockdown efficiency of AAV9-shUCP2 4 weeks after delivery of virus. n = 4 samples per group. ^*∗∗*^*P*<0.01 vs. the indicated group. (b) Representative images and results of echocardiographic assessment 12 h after intraperitoneal injection of PBS or LPS in AAV9-NC or AAV9-shUCP2 mice (n = 5 for each group). Data were presented as means ± SEM. ^*∗*^*P* < 0.05, ^*∗∗*^*P* < 0.01, and ^*∗∗∗*^*P* < 0.001 vs. the indicated group. (c) Survival of AAV9-NC (n = 8 for PBS, n = 12 for LPS) or AAV9-shUCP2 (n = 8 for PBS, n = 12 for LPS) mice intraperitoneally injected with PBS or LPS, monitored every 3 h for a 60-h period. Data were presented as means ± SEM. ^*∗∗∗*^*P* < 0.001 vs. the AAV-NC+PBS group; ^###^*P* < 0.001 vs. the AAV-NC+LPS group.

We subsequently sought to examine whether reduced levels of UCP2 in the heart attenuate septic cardiac dysfunction. To address this hypothesis, we chose a gene transfer approach using adenoassociated virus serotype 9 (AAV9), which shows strong tropism for cardiac myocytes. An AAV9 encoding shRNA specific for UCP2 (AAV9-shUCP2) or its control was injected intravenously into tail veins of mice, and 4 weeks later, LPS injection was performed. Mice treated with AAV9-shUCP2 showed a significant decrease in UCP2 protein expression (Figure 2(a)). At baseline, the AAV9-shUCP2 delivered mice exhibited a normal cardiac phenotype and cardiac function (data not shown). Remarkably, suppression of UCP2 expression with AAV9-shUCP2 significantly restored cardiac function (Figure 2(b)). Importantly, LPS-reduced lower survival rate in mice was significantly improved with AAV9-shUCP2 (Figure 2(c)). Taken together, these data indicate that UCP2 deletion prevented LPS-induced cardiac dysfunction and subsequent lethality.

### 3.3. Knockdown of UCP2 Preserved Mitochondrial Function in Cardiomyocytes

**Figure 3:**
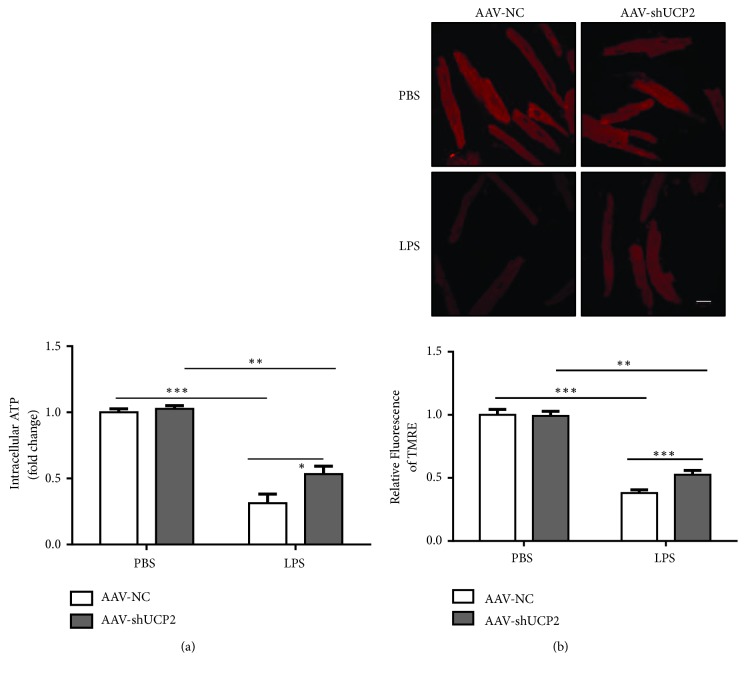
Knockdown of UCP2 preserved mitochondrial function in cardiomyocytes. (a) The intracellular ATP levels in the hearts 12 h after intraperitoneal injection of PBS or LPS in AAV9-NC or AAV9-shUCP2 mice. (b) Mitochondrial membrane potential, indicated by TMRE fluorescence of adult mice cardiomyocytes freshly isolated 12 h after intraperitoneal injection of PBS or LPS in AAV9-NC or AAV9-shUCP2 mice. ^*∗*^*P* < 0.05, ^*∗∗*^*P* < 0.01, and ^*∗∗∗*^*P* < 0.001 vs. the corresponding group. All data represent the mean ± SEM from at least 3 independent experiments.

ATP production is an important function of mitochondria, and its disorder is related with many cardiovascular diseases including heart failure [22]. Therefore, we examined the intracellular ATP levels in mice hearts. Our data suggest that knockdown of UCP2 significantly improved the reduced levels of intracellular ATP in the LPS-insulted myocardium (Figure 3(a)).

Processes interfering with maintenance of mitochondrial membrane potential may impact on cellular energy supply and mitochondrial ROS production. Next, we isolated primary adult mouse cardiomyocytes with or without LPS injection. LPS treatment induced a significant decline in TMRE fluorescence (Figure 3(b)), indicating a decline in mitochondrial membrane potential, which was improved by UCP2 knockdown with AAV9-shUCP2. Those data suggest that UCP2 could protect LPS-induced mitochondrial damage and preserve mitochondrial function during septic cardiac dysfunction.

### 3.4. UCP2 Promoted Autophagy in Response to LPS-Induced Sepsis

**Figure 4:**
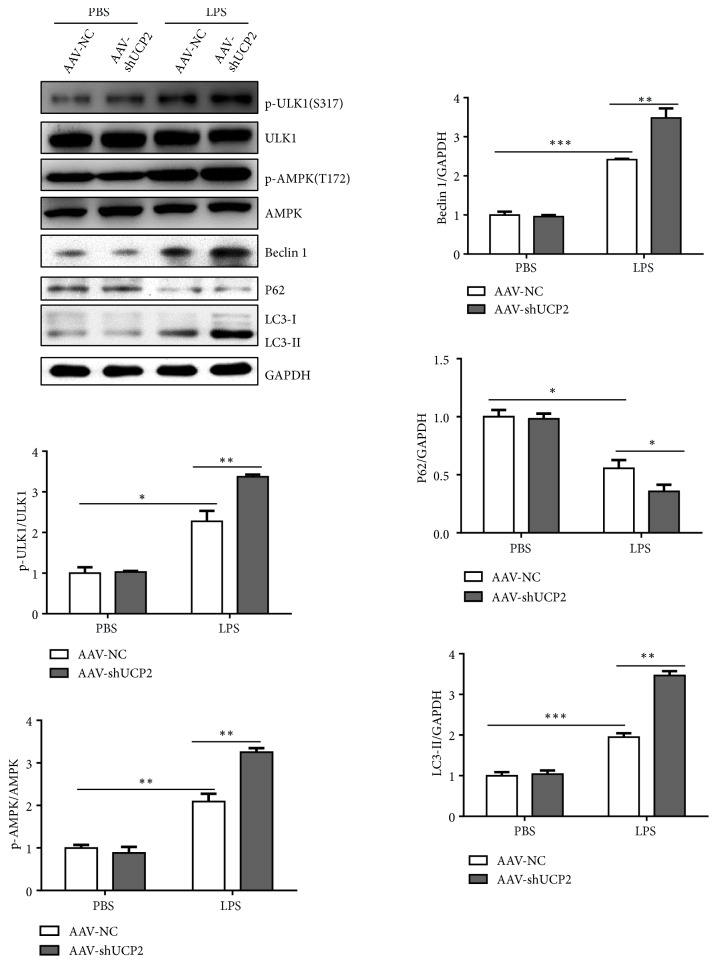
UCP2 knockdown activated AMPK-ULK1 signaling pathway and enhanced LPS-induced changes of cardiac autophagy. Representative immunoblots (upper panel) and quantitative analysis (lower panel) of AMPK, ULK1, light chain-3 (LC3), P62, and beclin 1 in LPS-challenged AAV9-NC or AAV9-shUCP2 mice, using GAPDH as a loading control in heart tissue lysates. Data were presented as means ± SEM. ^*∗*^*P* < 0.05, ^*∗∗*^*P* < 0.01, and ^*∗∗∗*^*P* < 0.001 vs. the corresponding group.

**Figure 5:**
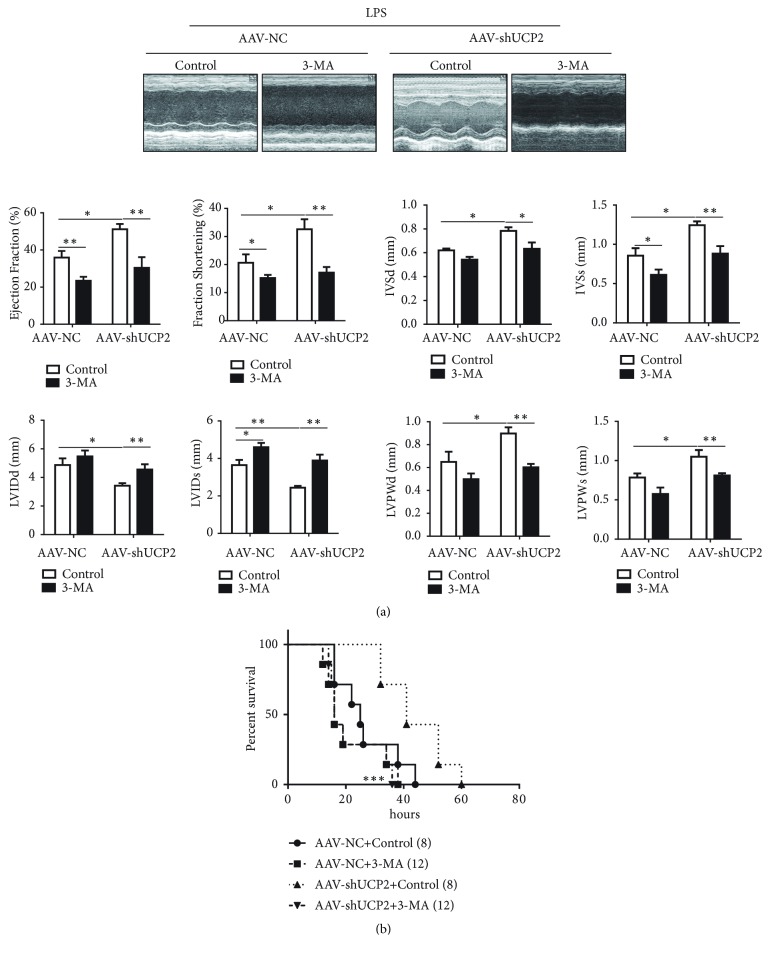
3-Methyladenine abolished the protective role of UCP2 knockdown in LPS-challenged mice. (a-b) WT and UCP2 knockdown mice were insulted with LPS i.p. 3-methyladenine was administered i.p. 30 minutes after LPS challenge. Cardiac function was measured 18 hours after LPS challenge. Representative images and results of echocardiographic assessment (n = 5 for each group). Data were presented as means ± SEM. ^*∗*^*P* < 0.05, ^*∗∗*^*P* < 0.01, and ^*∗∗*^*P* < 0.01 vs. the indicated group. (c) Survival was monitored in LPS (10 mg/kg) challenged UCP2 knockdown with or without 3-MA mice. ^*∗∗∗*^*P* < 0.001 vs. the AAV-shUCP2+Control group.

Several studies indicate that sepsis triggers autophagy in multiple organs including the heart and giving evidence that autophagy is an adaptive response. Mice were given either LPS or vehicle PBS and heart tissue was harvested after challenge. LC3II and beclin 1, markers of autophagosome formation, were examined by Western blot. Compared to shams, the level of LC3II and beclin 1 were higher in LPS-challenged animals, which was further increased with LPS insulted UCP2-knockdown mice (Figure 4). Consistent with an increase in autophagy, p62/SQSTM1, a polyubiquitin-binding protein that is degraded during autophagy showed the reverse trend (Figure 5). These data indicate that LPS stimulates cardiac autophagy activity, which was further enhanced by UCP2 knockdown.

To elucidate the signaling pathway involved in the UCP2-regulated autophagy, we examined regulation of the AMPK which induces autophagy by direct activating phosphorylation of ULK1 under conditions of energetic stress. Our results showed that AMPK/ULK1 pathway is significantly activated in LPS-challenged mice, which is further enhanced in UCP2-knockdown mice (Figure 4). Those data indicate that AMPK/ULK1 pathway is likely to be responsible for UCP2 knockdown-simulated autophagy.

### 3.5. Autophagy Inhibition Abolished the Protective Role of UCP2 Knockdown in LPS-Induced Sepsis

To examine the role of UCP2 regulated autophagy in cardiac dysfunction after sepsis, intraperitoneal injections of autophagy inhibitor 3-methyladenine (3-MA, 10 mg/kg) were applied to suppress autophagy. As anticipated, 3-MA delivery significantly decreased autophagy level in myocardium (data not shown). We examined heart performance by echocardiography in sham and LPS-challenged WT mice, UCP2 knockdown with and without 3-MA treated mice. LPS challenge substantially reduced EF, FS, and survival rate after LPS challenge in WT mice, which were preserved with UCP2 knockdown (Figure 5). However, 3-MA delivery abolished UCP2 knockdown-afforded cardioprotective effects (Figure 5). These data indicate that activated autophagy was responsible for the protective role of UCP2 knockdown in LPS-induced sepsis.

## 4. Discussion

Sepsis, a systemic inflammatory response syndrome caused by infection, is a serious threat to the lives of patients [23]. Sepsis can cause tissue hypoperfusion and septic shock which leads to organ dysfunction and death via a variety of mechanisms. This investigation was aimed at determining the role of UCP2 in the heart during sepsis and to test the therapeutic potential of targeting this gene with a cardiotropic adenoassociated viral vector encoding shRNA for UCP2 using a mouse model of LPS-induced sepsis. We found that LPS triggered a significantly increased expression of UCP2 followed by induced autophagic response. Then UCP2 knockdown* in vivo* further activated autophagy and improved cardiac function after LPS insult, which abrogated with autophagy inhibitor 3-MA. These novel findings clearly suggest that UCP2-regulated autophagy might play a crucial role in the process of sepsis, which possesses a therapeutic potential for sepsis.

Previous study reported that UCP2 in blood cells of sepsis patients was significantly higher than that of healthy controls and the expression level of UCP2 in blood cells of sepsis patients was significantly reduced after treatment, compared to that before treatment [24]. UCP2 level in blood cells might be a specific biomarker for sepsis, which is positively correlated with the severity of sepsis. However, the present study was designed to investigate the level of UCP2 in myocardium and examine its roles in septic cardiac dysfunction. Our studies indicated that UCP2 mRNA and protein level are remarkably increased in myocardium. Most importantly, UCP2 knockdown evokes resistance to LPS-induced septic cardiac dysfunction and lethality* in vivo*. In the meanwhile, UCP2 deficiency preserved the reduced levels of intracellular ATP in the heart tissues and improved mitochondrial membrane potential in adult mouse cardiomyocytes in LPS-induced septic mice. We therefore conclude that ablating UCP2 in myocardial has a marked protective effect against septic cardiac dysfunction.

Autophagy presents a dominant role in inflammation by limiting the availability of inflammasome activators[25], and by reducing mitochondrial DAMPs through activation of mitophagy [26]. However, excessive increase in autophagy due to deregulated degradation of cellular components resulted in aggressive inflammation [27]. Previous studies have used pharmacological approaches to examine the role of autophagy in sepsis, which come to the opposite conclusion [28, 29] Evidence obtained* in vivo* using a mouse cercal ligation and puncture (CLP) sepsis model [30] and in vitro using cultured, lipopolysaccharide- (LPS-) challenged cardiomyocytes [31], suggests that stimulating autophagy via pharmacological approaches protects the myocardium, thus providing evidence that autophagy is an adaptive response. On the contrary, recent paper showed that reducing autophagy improved cardiac function in a mouse model of LPS induced sepsis, supporting the conclusion that autophagy is maladaptive [32]. The contradictory results may be interpreted by the differences in experimental settings, in which the severity of sepsis, drug specificity, and timing of delivery differed. In this report, we observed that autophagy activity is elevated (Figure 4), which is an adaptive response and too mild to carry deterrent value under LPS challenge. Then UCP2 knockdown further activated autophagy and protected cardiac function and improved survival rate during sepsis. Accumulated evidence has indicated that under different nutritional statuses AMPK is coordinated closely with mTOR in regulating autophagy through direct phosphorylation of ULK1. To address molecular signaling pathway underlying the UCP2-modulated autophagy, changes in the autophagy trigger ULK1 and its upstream effectors, AMPK, were examined. Our data showed that AMPK/ULK1 signaling pathway is activated with LPS treatment and further enhanced after knockdown of UCP2. However, more experiments are needed to confirm this conclusion and how does UCP2 regulate AMPK/ULK1 signaling pathway. Our result suggests a signaling pathway from UCP2 to autophagy that protects cardiac mitochondria and cardiac function during sepsis.

## 5. Conclusion

Taken together, our findings revealed that UCP2 is particularly expressed in septic myocardium and essential to mediate LPS-induced cardiac injury, which pave the way towards future therapeutic approaches.

## Data Availability

The data used to support the findings of this study are available from the corresponding author upon request.
